# On the inverse problem of vibro-acoustography

**DOI:** 10.1007/s11012-022-01485-w

**Published:** 2022-02-28

**Authors:** Barbara Kaltenbacher

**Affiliations:** University of Klagenfurt, Klagenfurt, Austria

**Keywords:** Vibro-acoustic imaging, Inverse problem, Coefficient identification, Regularization, 35R30, 65J20

## Abstract

The aim of this paper is to put the problem of vibroacoustic imaging into the mathematical framework of inverse problems (more precisely, coefficient identification in PDEs) and regularization. We present a model in frequency domain, prove uniqueness of recovery of the spatially varying nonlinearity parameter from measurements of the acoustic pressure at multiple frequencies, and derive Newton as well as gradient based reconstruction methods.

## Introduction

Vibro-acoustography by means of ultrasound was developed [1, 2] to achieve the high resolution by high frequency waves while avoiding the drawbacks of scattering from small inclusions and of stronger attenuation at higher frequencies.

**Fig. 1:**
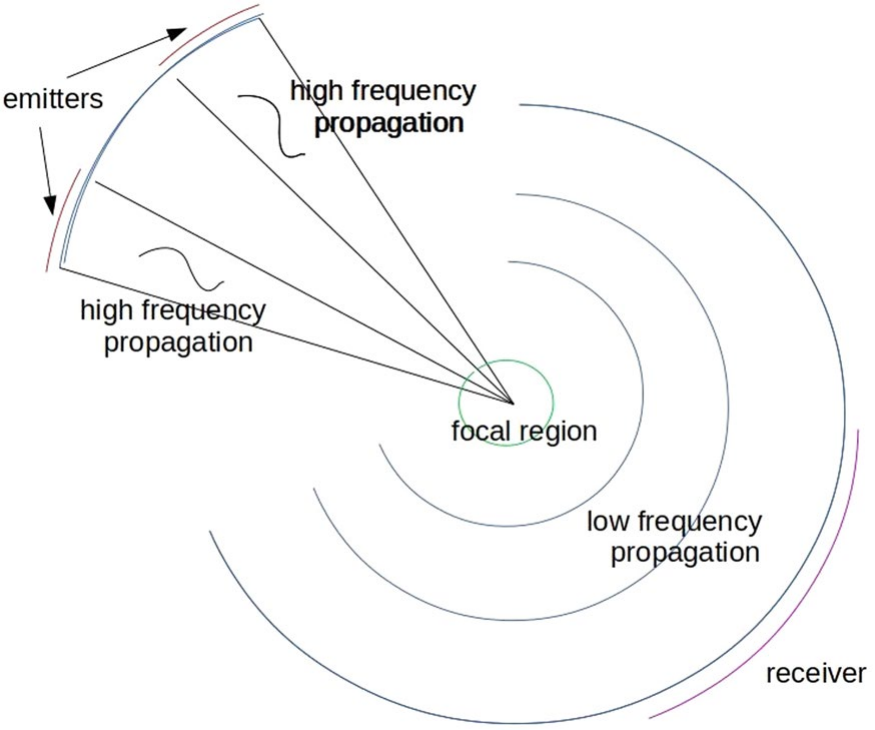
schematic of the experimental setup

The experiment for image acquisition is schematically illustrated in Fig. 1: Two ultrasound beams of high and slightly different frequencies $\omega _1$ and $\omega _2$ are excited at two parts $\Sigma _1$, $\Sigma _2$ of an array of piezoelectric transducers (emitters). They interact nonlinearly at a focus and this interaction excites a wave that basically propagates at the difference frequency $\omega _1-\omega _2$ and is eventually measured by a receiver array $\Gamma$ (in experiments consisting of hydrophones, in imaging this would be a piezoelectric transducer array as well). After each measurement, the focal region is shifted to scan the overall region of interest. Inhomogeneity of the medium leads to spatial dependence of two coeffients in the governing models: The speed of sound $c=c(x)$ and the nonlinearity parameter $\gamma =\gamma (x)$. Both parameters are susceptible to local variations in the acoustic medium (e.g., human tissue in medical applications) and thus their reconstruction yields a spatial image of the region of interest. In case of reconstructing the individual coefficients $c=c(x)$ or $\gamma =\gamma (x)$, this is related to ultrasound tomography and nonlinearity parameter imaging, respectively, cf. e.g., [3–6] and the citing literature.

A modeling and simulation framework for this methodology has been devised in [7, 8]. In this paper we put an emphasis on the inverse problem of reconstructing $c=c(x)$ and $\gamma =\gamma (x)$.

## Model

Two ultrasound beams with acoustic velocity potentials $\phi _1$, $\phi _2$ are excited by transducers and their interaction in turn excites a wave field with velocity potential $\psi$. This is described by a system of PDEs with inhomogeneous Neumann conditions

1 $$  \begin{aligned}   \partial _{t}^{2} \phi _{k}  - c^{2} \Delta \phi _{k}  = 0\;{\text{in}}\;\Omega , \hfill \\   \partial _{\nu } \phi _{k}  = g_{k} \;{\text{on}}\;\Sigma _{k} ,\quad k \in \left\{ {1,2} \right\} \hfill \\  \end{aligned}  $$

2 $$\begin{aligned}&\partial _t^2 \psi -c^2\Delta \psi = {\tilde{f}}(\phi _1,\phi _2,\gamma ,c) {\text{ in }} \Omega \nonumber \\&{\tilde{f}}(\phi _1,\phi _2,\gamma ,c)=\nonumber \\&\partial _t\left( \left| \nabla (\phi _1+\phi _2)\right| ^2 + \frac{\gamma -1}{2c^2} \left| \partial _t(\phi _1+\phi _2)\right| ^2\right) \end{aligned}$$

see [9, 10] for the derivation of the nonlinear forcing *f*. In here, $c=c(x)$ and $\gamma =\gamma (x)$ are the spatially varying sound speed and nonlinearity parameter, respectively, $\Omega \subseteq {\mathbb {R}}^d$, $d\in \{2,3\}$, and the manifold $\Sigma _k$ represents the emitting transducer array with given time harmonic excitation $g_k(x,t)={\hat{g}}_k(x)\, e^{\omega _k t}$.

The system (1), (2) is not fully nonlinear but the task of its solution can be decoupled into two linear subproblems: First compute $\phi _1,\phi _2$ from (1), then insert them into the right hand side of (2), and finally solve (2) for $\psi$.

Transformation into frequency domain Linearity of the subproblems allows to easily transfer the time domain formulation (1), (2) into frequency domain in case of a time harmonic excitation. With the time harmonic ansatz $\phi _k(x,t)={\hat{\phi }}_k(x)\, e^{\omega _k t}$, $\psi (x,t)=\Re \left({\hat{\psi }}(x)\, e^{(\omega _1-\omega _2)t}\right)$, where the latter is induced by real-valuedness of ${\tilde{f}}$ in (2)

$$\begin{aligned}&{\tilde{f}}(\phi _1,\phi _2,\gamma ,c)\\&\quad =\partial _t\Bigl [\left| \nabla {\hat{\phi }}_1\right| ^2+\left| \nabla {\hat{\phi }}_2\right| ^2 +2\Re \left( \nabla {\hat{\phi }}_1\cdot \nabla \overline{{\hat{\phi }}_2}\, e^{(\omega _1-\omega _2)t}\right) \\&\quad +\frac{\gamma (x)-1}{2c(x)^2}\Bigl (\left| \omega _1{\hat{\phi }}_1\right| ^2+\left| \omega _2{\hat{\phi }}_2\right| ^2\\&\quad +2\omega _1\omega _2\Re \left( {\hat{\phi }}_1\overline{{\hat{\phi }}_2}\, e^{(\omega _1-\omega _2)t}\right) \Bigr )\Bigr ]\\&=2(\omega _1-\omega _2)\\&\quad \cdot \Re \Bigl (\left( \nabla {\hat{\phi }}_1\cdot \overline{\nabla {\hat{\phi }}_2} +\omega _1\omega _2 \frac{\gamma (x)-1}{2c(x)^2}{\hat{\phi }}_1\overline{{\hat{\phi }}_2}\right) e^{(\omega _1-\omega _2)t}\Bigr ), \end{aligned}$$

we get

3 $$\begin{aligned}&-\frac{\omega _k^2}{c(x)^2} {\hat{\phi }}_k-\Delta {\hat{\phi }}_k=0 {\text{ in }} \Omega \nonumber \\&\partial _\nu {\hat{\phi }}_k={\hat{g}}_k {\text{ on }} \Sigma _k\,,\quad k\in \{1,2\} \end{aligned}$$

4 $$\begin{aligned}&-\frac{(\omega _1-\omega _2)^2}{c(x)^2} {\hat{\psi }}-\Delta {\hat{\psi }}= f({\hat{\phi }}_1,{\hat{\phi }}_2,\gamma ,c) {\text{ in }} \Omega \quad \nonumber \\&f({\hat{\phi }}_1,{\hat{\phi }}_2,\gamma ,c)=\frac{2(\omega _1-\omega _2)}{c(x)^2}\nonumber \\&\cdot \left( \nabla {\hat{\phi }}_1\cdot \overline{\nabla {\hat{\phi }}_2} +\omega _1\omega _2 \frac{\gamma (x)-1}{2c(x)^2}{\hat{\phi }}_1\overline{{\hat{\phi }}_2}\right) \end{aligned}$$

which nicely illustrates the physical fact that the propagating wave described by $\psi$ is concentrated at the difference frequency $\omega _1-\omega _2$.

We mention in passing that in fact also in the harmonic ansatz for $\phi _1$, $\phi _2$ taking the real part would be demanded by physics. This would lead to certain (actually higher frequency) correction terms, that we neglect here, though, as they are not relevant for reconstructions. As a consequence, (3), (4) is not fully equivalent to the equation we would get – via Fourier transform in time under an $L^2({\mathbb {R}})$ assumption—from (1), (2).

Boundary conditions We consider a bounded computational domain $\Omega$, where the excitation surfaces $\Sigma _k$ are part of the boundary $\Sigma _k\subseteq \partial \Omega$ and the rest of $\partial \Omega$ is subject to impedance boundary conditions in order to damp reflected waves

$$\begin{aligned} \partial _\nu {\hat{\phi }}_k=-\sigma _k {\hat{\phi }}_k {\text{ on }} \partial \Omega \setminus \Sigma _k\,, \quad \partial _\nu {\hat{\psi }}=-\sigma {\hat{\psi }} {\text{ on }}\partial \Omega \,. \end{aligned}$$

with nonnegative $L^\infty$ impedance coefficients $\sigma ,\sigma _k$ that are bounded away from zero on an open subset of $\partial \Omega$ or $\partial \Omega \setminus \Sigma _k$, respectively.

Measurements The pressure data taken at the receiver array can, via the identity

$$\begin{aligned} \varrho \partial _t\psi =p \end{aligned}$$

where $\varrho$ is the mass density and *p* the pressure be expressed by an observation operator

5 $$\begin{aligned} C:({\hat{\phi }}_1, {\hat{\phi }}_2 ,{\hat{\psi }})\mapsto (\omega _1-\omega _2){\text{tr}}_\Gamma {\hat{\psi }} \end{aligned}$$

where $\Gamma$ is a manifold representing the receiver array and lying inside the acoustic domain $\Omega$ or on its boundary.

Inverse problem The inverse problem of vibro-acoustography consists of determining the spatially varying coefficients *c* and $\gamma$ from observations (5) of the low frequency wave field. We assume that *c* is known on the outer boundary and needs to be reconstructed only in a subdomain (region of interest) $\widetilde{\Omega }\subseteq \Omega$ of the computational domain. With a slight abuse of notation we write

$$\begin{aligned}\frac{1}{c^2}=:\underline{\widetilde{\kappa }}=\kappa _0+\chi _{\widetilde{\Omega }}\kappa \,, \quad \frac{\gamma -1}{2c^4}=:\underline{\widetilde{\gamma }}=\gamma _0+\chi _{\widetilde{\Omega }}\gamma \end{aligned}$$

where the background $\kappa _0$, $\gamma _0\in L^\infty (\Omega )$, $\kappa _0(x)\ge {\underline{\kappa }}>0$, and the subdomain ${\tilde{\Omega }}$ are known and $\chi _{\widetilde{\Omega }}$ is the extension by zero operator from $\widetilde{\Omega }$ to $\Omega$, defined by $(\chi _{\widetilde{\Omega }}\kappa )(x)=\kappa (x)$ for $x\in \widetilde{\Omega }$ and zero else. Therewith our aim is to recover $\kappa ,\gamma \in L^2(\widetilde{\Omega })$, in the weak form of (3), (4)

6 $$\begin{aligned} \begin{aligned}&0=\langle A({\hat{\phi }}_1,{\hat{\phi }}_2,{\hat{\psi }},\kappa ,\gamma ),(v_1,v_2,w)\rangle :=\\&\Re \Bigl ((1-)\Bigl (\sum _{k=1}^2\int _{\Omega } (-\omega _k^2\underline{\widetilde{\kappa }}{\hat{\phi }}_k{\overline{v}}_k+\nabla {\hat{\phi }}_k\cdot \nabla {\overline{v}}_k)\, dx\\&+\int _{\Omega } (-(\omega _1-\omega _2)^2\underline{\widetilde{\kappa }}{\hat{\psi }}{\overline{w}}+\nabla {\hat{\psi }}\cdot \nabla {\overline{w}})\, dx\\&+\int _{\partial \Omega \setminus \Sigma _k}\sigma _k{\hat{\phi }}_k {\overline{v}}_k \, ds -\int _{\Sigma _k} {\hat{g}}_k {\overline{v}}_k\, ds +\int _{\partial \Omega }\sigma {\hat{\psi }} {\overline{w}} \, ds\\&-2(\omega _1-\omega _2) \\&\quad \cdot \int _\Omega \left( \underline{\widetilde{\kappa }}\nabla {\hat{\phi }}_1\cdot \overline{\nabla {\hat{\phi }}_2} +\omega _1\omega _2 \underline{\widetilde{\gamma }}{\hat{\phi }}_1\overline{{\hat{\phi }}_2}\right) \,{\overline{w}}\, dx \Bigr )\Bigr )\\&\text{ for } \text{ all } v_1,v_2,w\in H^1(\Omega ;{\mathbb {C}}) \end{aligned} \end{aligned}$$

(it suffices to take the real part here since $v_1,v_2,w$ vary over complex valued functions). This is the weak form of

7 $$\begin{aligned}&-\omega _k^2\underline{\widetilde{\kappa }}{\hat{\phi }}_k-\Delta {\hat{\phi }}_k=0 {\text{ in }} \Omega \nonumber \\&\partial _\nu {\hat{\phi }}_k={\hat{g}}_k {\text{ on }} \Sigma _k\,,\quad k\in \{1,2\} \end{aligned}$$

8 $$\begin{aligned}&-(\omega _1-\omega _2)^2\underline{\widetilde{\kappa }}{\hat{\psi }}-\Delta {\hat{\psi }}=f({\hat{\phi }}_1,{\hat{\phi }}_2,\underline{\widetilde{\kappa }},\underline{\widetilde{\gamma }}) {\text{ in }} \Omega \nonumber \\&\quad f({\hat{\phi }}_1,{\hat{\phi }}_2,\underline{\widetilde{\kappa }},\underline{\widetilde{\gamma }})=2(\omega _1-\omega _2) \nonumber \\&\cdot \left(\underline{\widetilde{\kappa }}\nabla {\hat{\phi }}_1\cdot \overline{\nabla {\hat{\phi }}_2} +\omega _1\omega _2 \underline{\widetilde{\gamma }}{\hat{\phi }}_1\overline{{\hat{\phi }}_2}\right) \end{aligned}$$

with homogeneous impedance boundary conditions on (the rest of) $\partial \Omega$, which we also tacitly assume to hold in the following.

In Section 3 we will prove that for every $\kappa ,\gamma \in L^2(\widetilde{\Omega })$, there exists a unique solution ${\hat{\phi }}_1,{\hat{\phi }}_2,{\hat{\psi }}$ of the operator equation $A({\hat{\phi }}_1,{\hat{\phi }}_2,{\hat{\psi }},\kappa ,\gamma )=0$ in appropriate function spaces, such that also the observation operator *C* according to (5) can be applied and yields an element of $L^2(\Gamma )$.

This justifies the use of the function spaces

9 $$\begin{aligned} X=L^2(\widetilde{\Omega })\times L^2(\widetilde{\Omega })\,, \quad Y=L^2(\Gamma ), \end{aligned}$$

to define the forward operator

10 $$\begin{aligned} &F:X\rightarrow Y, \quad  \left[{\begin{array}{c}\kappa \gamma \end{array}} \right]\mapsto F( \left[{\begin{array}{c}\kappa \gamma \end{array}} \right])=(\omega _1-\omega _2){\text{tr}}_\Gamma {\hat{\psi }}\\&{\text{ where }} \;{\hat{\phi }}_1,{\hat{\phi }}_2,{\hat{\psi }}\;{\text{ solve }} \end{aligned} $$

and write the inverse problem in reduced form as

11 $$\begin{aligned} F( \left[{\begin{array}{c}\kappa \gamma \end{array}} \right])=y \end{aligned}$$

where $y\in L^2(\Gamma )$ is the pressure distribution measured at the receiver array. Concerning the choice of spaces (9), working in $L^2$ spaces makes definition of methods most convenient. This is on one hand due to their Hilbert space structure, on the other hand due to the fact that no derivatives are involved, which avoids having to solve additional PDEs for evaluating the adjoint operator.

Alternatively, using the model and observation operators *A* and *C* defined in (5), (6), we may write the inverse problem as an all-at-once system for the parameters $$\left[{\begin{array}{c}\kappa \gamma \end{array}} \right]$$ and the states $u:=({\hat{\phi }}_1,{\hat{\phi }}_2,{\hat{\psi }})$ as

12 $$\begin{aligned} \begin{aligned}&A\left( u, \left[{\begin{array}{c}\kappa \gamma \end{array}} \right]\right) =0\\&Cu=y \end{aligned} \end{aligned}$$

The two formulations are related via the identity $F=C\circ S$, where the parameter-to-state map $$S: \left[{\begin{array}{c}\kappa \gamma \end{array}} \right]\rightarrow u:=({\hat{\phi }}_1,{\hat{\phi }}_2,{\hat{\psi }})$$ is implicitly defined by the identity

13 $$\begin{aligned} A\left( S( \left[{\begin{array}{c}\kappa \gamma \end{array}} \right]), \left[{\begin{array}{c}\kappa \gamma \end{array}} \right]\right) =0\,. \end{aligned}$$

Due to the fact that the forward problem is essentially linear (it amounts to sequentially solving a pair of linear homogeneous PDEs and another linear inhomogeneous one), the differences between all-at-once and reduced formulations are minor. Thus we will focus on the reduced setting (11) in the following.

## Forward problem and function space setting

In the following, function spaces such as $L^2(\Omega ;{\mathbb {C}})$ or $H^1(\Omega ;{\mathbb {C}})$ will be regarded as spaces of functions with values in ${\mathbb {C}}$, but treated as real Hilbert spaces with a real valued inner product, e.g. $(v,w)_{L^2(\Omega )}=\Re (\int _\Omega v{\overline{w}}\, dx)$. The $L^2$ space of real valued functions will simply be denoted by $L^2(\Omega )$.

Consider the Laplace operator equipped with impedance boundary conditions, defined in its weak form by

$$\begin{aligned} \begin{aligned}&\langle D_\sigma ({\hat{\psi }}),w\rangle :=B_\sigma ({\hat{\psi }},w)\\&:= \Re \left((1-)\left(\int _{\Omega } \nabla {\hat{\psi }}\cdot \nabla {\overline{w}}\, dx+\int _{\partial \Omega }\sigma {\hat{\psi }} {\overline{w}} \, ds\right)\right), \\&\forall w\in H^1(\Omega )\,. \end{aligned} \end{aligned}$$

Here $B_\sigma$ is a symmetric, bounded and coercive bilinear form on $H^1(\Omega ;{\mathbb {C}})$ by the identity

$$\begin{aligned} B_\sigma ({\hat{\psi }},{\hat{\psi }})= \int _{\Omega } \vert \nabla {\hat{\psi }}\vert ^2\, dx+\int _{\partial \Omega }\sigma \vert {\hat{\psi }}\vert ^2 \, ds \end{aligned}$$

and Poincaré’s inequality. Thus, by the Lax-Milgram Lemma, $D_\sigma :H^1(\Omega ;{\mathbb {C}})\rightarrow H^1(\Omega ;{\mathbb {C}})^*$ is boundedly invertible and its inverse is compact as an operator from $L^2_{{\tilde{\kappa }}_0}(\Omega ;{\mathbb {C}})$ into itself, where $L^2_{{\tilde{\kappa }}_0}(\Omega ;{\mathbb {C}})$ is the weighted $L^2$ space with weight function $\underline{\widetilde{\kappa }}\in L^2(\Omega )$, $\underline{\widetilde{\kappa }}\ge {\underline{\kappa }}>0$ a.e., so that $H^1(\Omega ;{\mathbb {C}})$ compactly embeds into $L^2_{{\tilde{\kappa }}_0}(\Omega ;{\mathbb {C}})$ and by $\Vert v\Vert _{(H^1)^*}\le \frac{1}{\sqrt{{\underline{\kappa }}}}\Vert v\Vert _{L^2_{{\tilde{\kappa }}_0}}\sup _{w\in H^1\setminus \{0\}}\frac{\Vert w\Vert _{L^2}}{\Vert w\Vert _{H^1}}$, also $L^2_{{\tilde{\kappa }}_0}(\Omega ;{\mathbb {C}})$ continuously embeds into $H^1(\Omega ;{\mathbb {C}})^*$. Thus, by spectral theory for compact operators, $D_\sigma$ has a countable sequence of positive real eigenvalues tending to infinity, which we will denote by $\{\lambda ^\sigma _n\, : n\in {\mathbb {N}}\}$. Likewise, the eigenvalues of the operators $D_{\sigma _k}$ defined by the Laplacian on $\Omega$ with impedance boundary conditions (coefficient $\sigma _k$) on $\partial \Omega \setminus \Sigma _k$ are given by the countable set $\{\lambda ^{\sigma _k}_n\, : n\in {\mathbb {N}}\}$, $k\in \{1,2\}$. Thus (6) (with ${\tilde{\kappa }}_0$ in place of ${\tilde{\kappa }}$) is uniquely solvable provided ${\hat{g}}_k\in H^{-\frac{1}{2}}(\Sigma _k)$ and $\omega _k\notin \{\lambda ^{\sigma _k}_n\, : n\in {\mathbb {N}}\}$, $k\in \{1,2\}$, $\omega _1-\omega _2\notin \{\lambda ^\sigma _n\, : n\in {\mathbb {N}}\}$, and we get well-definedness of the parameter-to-state map $S:{\mathcal {D}}_0(S)\rightarrow H^1(\Omega ;{\mathbb {C}})^3$ on ${\mathcal {D}}_0(S)=$ $\{\kappa \in L^2(\widetilde{\Omega })\, : \, \underline{\widetilde{\kappa }}\ge {\underline{\kappa }}>0 {\text{ a.e. }} \}\times L^2(\widetilde{\Omega })$. However, ${\mathcal {D}}_0(S)$ has empty interior with respect to the $L^2$ topology and this prevents applicability of convergence results for the Newton and gradient methods to be discussed below. To avoid this, we restrict *S* to an open ball around a strictly positive $L^\infty$ function ${\tilde{\kappa }}_0\ge {\underline{\kappa }}>0$ (e.g., the background $\kappa _0$)

14 $$\begin{aligned} \begin{aligned} {\mathcal {D}}(F)=&\{\kappa \in L^2(\widetilde{\Omega })\, : \, \Vert \underline{\widetilde{\kappa }}-{\tilde{\kappa }}_0\Vert _{L^2}\le \rho \}\\&\times L^2(\widetilde{\Omega }) \end{aligned} \end{aligned}$$

for $\rho$ sufficiently small, and apply a fixed point argument to obtain well-definedness of *S* on ${\mathcal {D}}(F)$, see, e.g., [11].

Higher regularity (actually only higher summability) can be achieved under the additional assumption ${\hat{g}}_k\in (W^{1-\frac{1}{q},\frac{q}{q-1}}(\partial \Omega \setminus \Sigma _k))^*\subseteq ({\text{tr}}_{\Sigma _k}(W^{1,\frac{q}{q-1}}(\Omega )))^*$, meaning that the linear map $v\mapsto \int _\Sigma {\hat{g}}_k v\, ds$ lies in $(W^{1,\frac{q}{q-1}}(\Omega )))^*$. Therefore according to elliptic regularity (e.g. [12, Theorem 7.7]), (7) admits weak solutions ${\hat{\phi }}_k\in W^{1,q}(\Omega ;{\mathbb {C}})$, $k\in \{1,2\}$. Thus, the right hand side of (8) has the following regularity. From $\underline{\widetilde{\kappa }}\in L^2(\Omega )$ and $\nabla {\hat{\phi }}_1$, $\overline{\nabla {\hat{\phi }}_2} \in L^{q}(\Omega )$ we conclude by Hölder’s inequality

15 $$\begin{aligned} \begin{aligned}&\Vert a\,b\,{\overline{c}}\Vert _{L^r}\le \Vert a\Vert _{L^2}\Vert b\Vert _{L^{\frac{4r}{2-r}}}\Vert c\Vert _{L^{\frac{4r}{2-r}}}\\&{\text{ for}}\; {\rm {any }}  a\in L^2(\Omega ), \ b,\,c\in L^{\frac{4r}{2-r}}(\Omega ,{\mathbb {C}}) \end{aligned} \end{aligned}$$

that $$\underline{\widetilde{\kappa }}\nabla {\hat{\phi }}_1\cdot \overline{\nabla {\hat{\phi }}_2} \in L^r(\Omega )\subseteq W^{-1,p}(\Omega )$$, provided

16 $$\begin{aligned} r\le \min \left\{ 2,\frac{q}{2}\right\} {\text{ and }} \frac{2r}{2-r}\le \frac{q}{2} {\text{ and }} 1-\frac{d}{p^*}\ge -\frac{d}{r^*} \end{aligned}$$

where $p^*=\frac{p}{p-1}$ denotes the dual index. This regularity (and even more) also holds true for the second quadratic term $\underline{\widetilde{\gamma }}{\hat{\phi }}_1\overline{{\hat{\phi }}_2}$, with $\underline{\widetilde{\gamma }}\in L^2(\Omega )$. Thus we conclude $\psi \in W^{1,p}(\Omega )$ (cf. [12, Theorem 7.7]) and hence, by the Trace Theorem, ${\text{tr}}_\Gamma \psi \in W^{1-\frac{1}{p},p}(\Gamma )\subseteq L^2(\Gamma )$ provided

17 $$\begin{aligned} 1-\frac{1}{p}-\frac{d-1}{p}\ge -\frac{d-1}{2}\,. \end{aligned}$$

It is readily checked that conditions (16), (17) can be satisfied for $\Omega \subseteq {\mathbb {R}}^d$, by choosing,

18 $$\begin{aligned} \begin{aligned}&\frac{2d}{d+2}\le p^*\le \frac{2d}{d-1}, \\&2\le r^*\le \frac{d p^*}{d-p^*} ({\text{ or }} p^*\ge d), \quad q\ge \frac{4r}{2-r}, \end{aligned} \end{aligned}$$

that is, in the physically relevant case $d\le 3$, e.g. $p=\frac{3}{2}$, $r=1$, $q=4$.

Thus we have proven

### Theorem 1

Let ${\tilde{\kappa }}_0\ge {\underline{\kappa }}>0$ a.e. and ${\hat{g}}_k\in H^{-\frac{1}{2}}(\Sigma _k)$ and $\omega _k\notin \{\lambda ^{\sigma _k}_n\, : n\in {\mathbb {N}}\}$, $k\in \{1,2\}$, $\omega _1-\omega _2\notin \{\lambda ^\sigma _n\, : n\in {\mathbb {N}}\}$ the sets of $L^2_{{\tilde{\kappa }}_0}$ eigenvalues of the Laplacians $D_{\sigma _k}$, $D_\sigma$ with impedance boundary conditions.

Then the parameter-to-state map $S:{\mathcal {D}}(F)\rightarrow W^{1,q}(\Omega ;{\mathbb {C}})^2\times W^{1,p}(\Omega ;{\mathbb {C}})$, with *p*, *q* as in (18) and the forward operator $F:{\mathcal {D}}(F)\rightarrow L^2(\Gamma )$ are well-defined by (5), (6), (10), (13) on ${\mathcal {D}}(F)$ as in (14) with $\rho >0$ sufficiently small.

For use in Newton and gradient type methods we also need differentiability of *F*. It sufficies to prove that the parameter-to-state map *S* is differentiable, since $F=C\circ S$ with *C* being a bounded linear operator. It is straightforward to see that for $$({\hat{\phi }}_1,{\hat{\phi }}_2,{\hat{\psi }}):=S( \left[{\begin{array}{c}\kappa \gamma \end{array}} \right])$$, $$({\hat{\phi }}_1^+,{\hat{\phi }}_2^+,{\hat{\psi }}^+):=S( \left[{\begin{array}{c}\kappa \gamma \end{array}} \right]+\delta  \left[{\begin{array}{c}\kappa \gamma \end{array}} \right])$$ the difference $$(d{\hat{\phi }}_1,d{\hat{\phi }}_2,d{\hat{\psi }}):=S( \left[{\begin{array}{c}\kappa \gamma \end{array}} \right]+\delta  \left[{\begin{array}{c}\kappa \gamma \end{array}} \right])-S( \left[{\begin{array}{c}\kappa \gamma \end{array}} \right])$$ satisfies the weak form of

19 $$\begin{aligned} \begin{aligned}&-\omega _k^2\underline{\widetilde{\kappa }}\, d{\hat{\phi }}_k-\Delta \, d{\hat{\phi }}_k=\omega _k^2\,\underline{\widetilde{\delta \kappa }}\, {\hat{\phi }}_k^+ \text{ in } \Omega \\&\quad \partial _\nu \, d{\hat{\phi }}_k=0 \text{ on } \Sigma _k\,,\quad k\in \{1,2\} \\&\quad -(\omega _1-\omega _2)^2\underline{\widetilde{\kappa }}\, d{\hat{\psi }}-\Delta \, d{\hat{\psi }}=f_d \text{ in } \Omega \\&\quad f_d= (\omega _1-\omega _2)^2\underline{\widetilde{\kappa }}\, {\hat{\psi }}^+ +2(\omega _1-\omega _2)\\&\quad \cdot \Bigl (\underline{\widetilde{\delta \kappa }}\,\nabla {\hat{\phi }}_1^+\cdot \overline{\nabla {\hat{\phi }}_2^+} +\underline{\widetilde{\kappa }}(\nabla \, d{\hat{\phi }}_1\cdot \overline{\nabla {\hat{\phi }}_2^+} +\nabla {\hat{\phi }}_1\cdot \overline{\nabla \, d{\hat{\phi }}_2})\\&\quad + \omega _1\omega _2 \left( \underline{\widetilde{\delta \gamma }}\,{\hat{\phi }}_1^+\overline{{\hat{\phi }}_2^+} +\underline{\widetilde{\gamma }}( d{\hat{\phi }}_1\overline{{\hat{\phi }}_2^+} +{\hat{\phi }}_1\,\overline{d{\hat{\phi }}_2}) \right) \Bigr ) \end{aligned} \end{aligned}$$

with $\underline{\widetilde{\delta \kappa }}=\chi _{\widetilde{\Omega }}\delta \kappa$, $\underline{\widetilde{\delta \gamma }}=\chi _{\widetilde{\Omega }}\delta \gamma$, and therefore, formally $$(\delta {\hat{\phi }}_1,\delta {\hat{\phi }}_2,\delta {\hat{\psi }}):=S'( \left[{\begin{array}{c}\kappa \gamma \end{array}} \right])\delta  \left[{\begin{array}{c}\kappa \gamma \end{array}} \right]$$ solves

20 $$\begin{aligned} \begin{aligned}&-\omega _k^2\underline{\widetilde{\kappa }}\, \delta {\hat{\phi }}_k-\Delta \, \delta {\hat{\phi }}_k=\omega _k^2\,\underline{\widetilde{\delta \kappa }}\, {\hat{\phi }}_k \text{ in } \Omega \\&\quad \partial _\nu \, \delta {\hat{\phi }}_k=0 \text{ on } \Sigma _k\,,\quad k\in \{1,2\} \\&\quad -(\omega _1-\omega _2)^2\underline{\widetilde{\kappa }}\, \delta {\hat{\psi }}-\Delta \, \delta {\hat{\psi }}= f_\delta \text{ in } \Omega \\&\quad f_\delta =(\omega _1-\omega _2)^2\underline{\widetilde{\kappa }}\, {\hat{\psi }} +2(\omega _1-\omega _2)\\&\quad \cdot \Bigl (\underline{\widetilde{\delta \kappa }}\,\nabla {\hat{\phi }}_1\cdot \overline{\nabla {\hat{\phi }}_2} +\underline{\widetilde{\kappa }}(\nabla \, \delta {\hat{\phi }}_1\cdot \overline{\nabla {\hat{\phi }}_2} +\nabla {\hat{\phi }}_1\cdot \overline{\nabla \, \delta {\hat{\phi }}_2})\\&\quad + \omega _1\omega _2 \left( \underline{\widetilde{\delta \gamma }}\,{\hat{\phi }}_1\overline{{\hat{\phi }}_2} +\underline{\widetilde{\gamma }}( \delta {\hat{\phi }}_1\overline{{\hat{\phi }}_2} +{\hat{\phi }}_1\,\overline{\delta {\hat{\phi }}_2}) \right) \Bigr )\,. \end{aligned} \end{aligned}$$

Hence the first order Taylor remainder $$(\check{\phi }_1,\check{\phi }_2,\check{\psi }):=S( \left[{\begin{array}{c}\kappa \gamma \end{array}} \right]+\delta  \left[{\begin{array}{c}\kappa \gamma \end{array}} \right])-S( \left[{\begin{array}{c}\kappa \gamma \end{array}} \right])-S'( \left[{\begin{array}{c}\kappa \gamma \end{array}} \right])\delta  \left[{\begin{array}{c}\kappa \gamma \end{array}} \right]$$ obeys

21 $$\begin{aligned} \begin{aligned}&-\omega _k^2\underline{\widetilde{\kappa }}\, \check{\phi }_k -\Delta \, \check{\phi }_k=\omega _k^2\,\underline{\widetilde{\delta \kappa }}\, d{\hat{\phi }}_k \text{ in } \Omega \\&\quad \partial _\nu \, \check{\phi }_k=0 \text{ on } \Sigma _k\,,\quad k\in \{1,2\}\\&\quad -(\omega _1-\omega _2)^2\underline{\widetilde{\kappa }}\, \check{\psi }-\Delta \, \check{\psi }=f_{rem } \text{ in } \Omega \\&\quad f_{rem }= (\omega _1-\omega _2)^2\underline{\widetilde{\kappa }}\, d{\hat{\psi }} +2(\omega _1-\omega _2)\\&\quad \cdot \Bigl (\underline{\widetilde{\delta \kappa }}\, (\nabla \, d{\hat{\phi }}_1\cdot \overline{\nabla {\hat{\phi }}_2^+} +\nabla {\hat{\phi }}_1\cdot \, \overline{d\nabla {\hat{\phi }}_2})\\&\quad +\underline{\widetilde{\kappa }}(\nabla \, \check{\phi }_1\cdot \overline{\nabla {\hat{\phi }}_2} +\nabla \, d{\hat{\phi }}_1\cdot \overline{\nabla \, d{\hat{\phi }}_2} +\nabla {\hat{\phi }}_1\cdot \overline{\nabla \check{\phi }_2})\\&\quad + \omega _1\omega _2 \Bigl ( \underline{\widetilde{\delta \gamma }}\, (d{\hat{\phi }}_1\overline{{\hat{\phi }}_2^+} +{\hat{\phi }}_1\, \overline{d{\hat{\phi }}_2})\\&\quad +\underline{\widetilde{\gamma }}(\check{\phi }_1\overline{{\hat{\phi }}_2} +\,d{\hat{\phi }}_1\,d\overline{{\hat{\phi }}_2} +{\hat{\phi }}_1\overline{\check{\phi }_2}) \Bigr )\Bigr ). \end{aligned} \end{aligned}$$

Here we have used the identities

$$\begin{aligned} \begin{aligned}&(a+\, \delta a) (b+db) (c+\,dc) - abc\\&\quad = \delta a\, (b+db)(c+\,dc)\, + \, a\, db \, (c+dc)\, +\, a\,b\,dc\\&\quad (a+\, \delta a) (b+db) (c+\,dc) - abc\\&\quad -(\delta a\, b\, c\, + \, a\, \delta b\, c\, +\, a\,b\,\delta c)\\&\quad =\delta a\,(db\, (c+dc)\, + \, b\, dc) \\&\qquad + a[(db-\delta b)c+db\,dc+b(dc-\delta c)]. \end{aligned} \end{aligned}$$

Regularity arguments as in the proof of Theorem 1 lead to estimates of the form

$$\begin{aligned} \begin{aligned}&\Vert d{\hat{\phi }}_k\Vert _{W^{1,q}}\le C \Vert \underline{\widetilde{\delta \kappa }}\, {\hat{\phi }}_k^+\Vert _{(W^{1,q^*})^*}\\&\Vert d{\hat{\psi }}\Vert _{W^{1,p}}\le C \Vert f_d\Vert _{(W^{1,p^*})^*}\\&\Vert \delta {\hat{\phi }}_k\Vert _{W^{1,q}}\le C \Vert \underline{\widetilde{\delta \kappa }}\, {\hat{\phi }}_k\Vert _{(W^{1,q^*})^*}\\&\Vert \delta {\hat{\psi }}\Vert _{W^{1,p}}\le C \Vert f_\delta \Vert _{(W^{1,p^*})^*}\\&\Vert \check{\phi }_k\Vert _{W^{1,q}}\le C \Vert \underline{\widetilde{\delta \kappa }}\, d{\hat{\phi }}_k\Vert _{(W^{1,q^*})^*}\\&\Vert \check{\psi }\Vert _{W^{1,p}}\le C \Vert f_{rem }\Vert _{(W^{1,p^*})^*}\,, \end{aligned} \end{aligned}$$

where $f_d$, $f_\delta$, $f_{rem }$ can be estimated by the same Hölder inequalities and Sobolev embeddings as those used for the proof of Theorem 1.

This proves Fréchet differentiability.

### Theorem 2

Under assumptions of Theorem 1, the parameter-to-state map *S* and the forward operator *F* are Fréchet differentiable on $\tilde{{\mathcal {D}}}(F)$ as defined in (14) with respect to the $L^2$ topology in preimage space, as mappings to $W^{1,q}(\Omega ;{\mathbb {C}})^2\times W^{1,p}(\Omega ;{\mathbb {C}})$ and $L^2(\Gamma )$, respectively.

Concerning further convergence conditions for Newton and gradient type methods, cf. e.g. [13], we briefly comment on the tangential cone condition

22 $$\begin{aligned} \begin{aligned}&\Vert F( \left[{\begin{array}{c}\kappa \gamma \end{array}} \right]+\delta  \left[{\begin{array}{c}\kappa \gamma \end{array}} \right])-F( \left[{\begin{array}{c}\kappa \gamma \end{array}} \right])-F'( \left[{\begin{array}{c}\kappa \gamma \end{array}} \right])\delta  \left[{\begin{array}{c}\kappa \gamma \end{array}} \right]\Vert \\&\quad \le c_{tc}\Vert F( \left[{\begin{array}{c}\kappa \gamma \end{array}} \right]+\delta  \left[{\begin{array}{c}\kappa \gamma \end{array}} \right])-F( \left[{\begin{array}{c}\kappa \gamma \end{array}} \right])\Vert . \end{aligned} \end{aligned}$$

In case of known speed of sound *c*, when we seek to identify $\gamma =\gamma (x)$ only, the inverse problem becomes an inverse source problem, see (29) below, and is therefore affinely linear, thus trivially satisfying (22) with $c_{tc}=0$. Conversely, if $c=c(x)$ is to be determined, the inverse probems is closely related to the well-known and well-investigated model problem of recovering the potential *c* in the Schrödinger equation $-\Delta u + c u=0$. This is known to satisfy the tangential cone condition only in case of complete observations of *u* on all of $\Omega$ [11]. Thus (22) cannot be expected to be verifiable in our boundary observation setting.

In the definition of gradient type methods (and also in the implementation of Newton type methods) we will need the adjoint of $$F'( \left[{\begin{array}{c}\kappa \gamma \end{array}} \right])$$, which we therefore derive here. First of all, note that by $F=C\circ S$ with *S* defined by (13) and the Implicit Function Theorem we can write

23 $$\begin{aligned} S'( \left[{\begin{array}{c}\kappa \gamma \end{array}} \right])=-K^{-1} L\,, \quad F'( \left[{\begin{array}{c}\kappa \gamma \end{array}} \right])=-CK^{-1} L\,, \end{aligned}$$

where

24 $$\begin{aligned} K=\frac{\partial A}{\partial u}(u^{(n)}, \left[{\begin{array}{c}\kappa \gamma \end{array}} \right]^{(n)})\,, \quad L=\frac{\partial A}{\partial  \left[{\begin{array}{c}\kappa \gamma \end{array}} \right]}(u^{(n)}, \left[{\begin{array}{c}\kappa \gamma \end{array}} \right]^{(n)}) \end{aligned}$$

are the linearizations of the operator *A* from (6) with respect to the states and the parameters, respectively. They are given by

25 $$\begin{aligned} \begin{aligned}&\langle K\, (\delta {\hat{\phi }}_1,\delta {\hat{\phi }}_2,\delta {\hat{\psi }}),(v_1,v_2,w)\rangle =\\&\quad \Re \Bigl ((1-)\Bigl (\sum _{k=1}^2\int _{\Omega } (-\omega _k^2\underline{\widetilde{\kappa }}\, \delta {\hat{\phi }}_k{\overline{v}}_k+\nabla \ \delta {\hat{\phi }}_k\cdot \nabla {\overline{v}}_k)\, dx\\&\quad +\int _{\Omega } (-(\omega _1-\omega _2)^2\underline{\widetilde{\kappa }}\, \delta {\hat{\psi }}{\overline{w}}+\nabla \, \delta {\hat{\psi }}\cdot \nabla {\overline{w}})\, dx\\&\quad +\int _{\partial \Omega \setminus \Sigma _k}\sigma _k\, \delta {\hat{\phi }}_k {\overline{v}}_k \, ds +\int _{\partial \Omega }\sigma \, \delta {\hat{\psi }} {\overline{w}} \, ds\\&\quad -\int _\Omega 2(\omega _1-\omega _2) \Bigl ( \underline{\widetilde{\kappa }}\Bigl (\nabla \, \delta {\hat{\phi }}_1\cdot \overline{\nabla {\hat{\phi }}_2} +\nabla {\hat{\phi }}_1\cdot \overline{\nabla \, \delta {\hat{\phi }}_2}\Bigr )\\&\quad +\omega _1\omega _2 \underline{\widetilde{\gamma }}\Bigl ( \, \delta {\hat{\phi }}_1\,\overline{{\hat{\phi }}_2} +{\hat{\phi }}_1\overline{\, \delta {\hat{\phi }}_2} \Bigr ) \Bigr )\Bigr )\,{\overline{w}}\, dx \Bigr ) \end{aligned} \end{aligned}$$

26 $$\begin{aligned} \begin{aligned}&\langle L\, (\delta \kappa ,\delta \gamma ),(v_1,v_2,w)\rangle =\\&\quad \Re \Bigl ((1-)\Bigl (\sum _{k=1}^2\int _{\Omega } -\omega _k^2 \underline{\widetilde{\delta \kappa }} \,{\hat{\phi }}_k{\overline{v}}_k\, dx\\&\quad +\int _{\Omega } -(\omega _1-\omega _2)^2 \underline{\widetilde{\delta \kappa }} \,{\hat{\psi }}{\overline{w}}\, dx\\&\quad -2(\omega _1-\omega _2) \\&\quad \cdot \int _\Omega \left( \underline{\widetilde{\delta \kappa }} \,\nabla {\hat{\phi }}_1\cdot \overline{\nabla {\hat{\phi }}_2} +\omega _1\omega _2 \, \underline{\widetilde{\delta \gamma }}\, {\hat{\phi }}_1\overline{{\hat{\phi }}_2}\right) \,{\overline{w}}\, dx \Bigr )\Bigr ) \end{aligned} \end{aligned}$$

for any $v_1,v_2,w\in H^1(\Omega ;{\mathbb {C}})$. The identity (23) with (24) can also be used to determine the adjoint operator $$F'( \left[{\begin{array}{c}\kappa \gamma \end{array}} \right])^*=-(CK^{-1}L)^*$$ as a Hilbert space adjoint in $L^2$. To this end, for given $r\in L^2(\Gamma )$ we want to find $$ \left[{\begin{array}{c}\xi \zeta \end{array}} \right]:=-(CK^{-1}L)^*r$$ such that

$$\begin{aligned} \begin{aligned}&\langle CK^{-1}L\,\delta  \left[{\begin{array}{c}\kappa \gamma \end{array}} \right],r\rangle _{L^2(\Gamma )} = -\langle \delta \kappa ,\xi \rangle _{L^2(\widetilde{\Omega })} -\langle \delta \gamma ,\zeta \rangle _{L^2(\widetilde{\Omega })} \\&\quad {\text{ for all }}  \delta  \left[{\begin{array}{c}\kappa \gamma \end{array}} \right]\in L^2(\widetilde{\Omega })\times L^2(\widetilde{\Omega })\,. \end{aligned} \end{aligned}$$

We introduce the auxiliary variables $$(\widetilde{\phi }_1,\widetilde{\phi }_2,\widetilde{\psi }):=-K^{-1}L\,\delta  \left[{\begin{array}{c}\kappa \gamma \end{array}} \right]$$, which allows us to use the identity

27 $$\begin{aligned} \begin{aligned}&\langle K(\widetilde{\phi }_1,\widetilde{\phi }_2,\widetilde{\psi }),(v_1,v_2,w)\rangle =-\langle L\delta  \left[{\begin{array}{c}\kappa \gamma \end{array}} \right],(v_1,v_2,w)\rangle \\&\quad {\text{ for all }}  v_1,v_2,w\in H^1(\Omega ;{\mathbb {C}}) \end{aligned} \end{aligned}$$

and define $(p_1,p_2,q)$ as the solution to the adjoint equation

28 $$\begin{aligned} \begin{aligned}&\langle K(\delta {\hat{\phi }}_1,\delta {\hat{\phi }}_2,\delta {\hat{\psi }}),(p_1,p_2,q)\rangle \\&\quad =\langle C(\delta {\hat{\phi }}_1,\delta {\hat{\phi }}_2,\delta {\hat{\psi }}),r\rangle _{L^2(\Gamma )} \\&\quad {\text{ for all }}  \delta {\hat{\phi }}_1,\delta {\hat{\phi }}_2,\delta {\hat{\psi }}\in H^1(\Omega ;{\mathbb {C}})\,. \end{aligned} \end{aligned}$$

Using (27), and (28) together with (25), (26), we get

$$\begin{aligned} \begin{aligned}&\langle C(\widetilde{\phi }_1,\widetilde{\phi }_2,\widetilde{\psi }),r\rangle _{L^2(\Gamma )} =-\langle L\,\delta  \bigl [ {\begin{array}{c}\kappa \gamma \end{array}} \bigr ],(p_1,p_2,q)\rangle \\&\quad =\Re \Bigl ((1-)\Bigl (\sum _{k=1}^2\int _{\widetilde{\Omega }} \omega _k^2 \,\delta \kappa \,{\hat{\phi }}_k^{(n)}{\overline{p}}_k\, dx\\&\quad +\int _{\widetilde{\Omega }} (\omega _1-\omega _2)^2 \,\delta \kappa \,{\hat{\psi }}^{(n)}{\overline{q}}\, dx\\&\quad +2(\omega _1-\omega _2) \\&\quad \cdot \int _{\widetilde{\Omega }} \left( \delta \kappa \,\nabla {\hat{\phi }}_1^{(n)}\cdot \overline{\nabla {\hat{\phi }}_2^{(n)}} +\omega _1\omega _2 \, \delta \gamma \, {\hat{\phi }}_1^{(n)}\overline{{\hat{\phi }}_2^{(n)}}\right) \,{\overline{q}}\, dx \Bigr )\Bigr )\\&\quad =\langle \delta \kappa ,\xi \rangle _{L^2(\widetilde{\Omega })} +\langle \delta \gamma ,\zeta \rangle _{L^2(\widetilde{\Omega })} \end{aligned} \end{aligned}$$

for

$$\begin{aligned} \begin{aligned}&\xi =\Re \Bigl ((1-)\Bigl (\sum _{k=1}^2 \omega _k^2 {\hat{\phi }}_k^{(n)}{\overline{p}}_k\\&\quad +(\omega _1-\omega _2)^2 {\hat{\psi }}^{(n)}{\overline{q}} +2(\omega _1-\omega _2) \nabla {\hat{\phi }}_1^{(n)}\cdot \overline{\nabla {\hat{\phi }}_2^{(n)}} {\overline{q}} \Bigr )\Bigr )\vert _{\widetilde{\Omega }} \\&\quad \zeta =\Re \Bigl ((1-)\Bigl (2(\omega _1-\omega _2) \omega _1\omega _2 {\hat{\phi }}_1^{(n)}\overline{{\hat{\phi }}_2^{(n)}}{\overline{q}}\Bigr )\Bigr )\vert _{\widetilde{\Omega }}. \end{aligned} \end{aligned}$$

Thus we end up with an explicit expression for $$ \left[{\begin{array}{c}\xi \zeta \end{array}} \right]:=F'( \left[{\begin{array}{c}\kappa \gamma \end{array}} \right])^*r$$. For this purpose, the adjoint states have to be computed as solutions to (28), that is, the weak form of

$$\begin{aligned} \begin{aligned}&-(\omega _1-\omega _2)^2\underline{\widetilde{\kappa }}q-\Delta q=0 {\text{ in }} \Omega \setminus \Gamma \,, \\&\quad \partial _\nu q=-\sigma q {\text{ on }} \partial \Omega \,, \quad \left[ \partial _\nu q\right] =(\omega _1-\omega _2) r {\text{ on }} \Gamma \end{aligned} \end{aligned}$$

where $\left[ \partial _\nu q\right]$ denotes the jump of the normal derivative over the interface $\Gamma$, as well as

$$\begin{aligned} \begin{aligned}&-\omega _k^2\underline{\widetilde{\kappa }}p_k-\Delta p_k =f_k {\text{ in }} \Omega \,, \\&\quad \partial _\nu p_k=-\sigma _k p_k {\text{ on }} \partial \Omega \setminus \Sigma _k\,, \quad \partial _\nu p_k=0 {\text{ on }} \Sigma _k\\&\quad f_k=-2(\omega _1-\omega _2)\left( \nabla \left( \underline{\widetilde{\kappa }}q\nabla \phi _{\not k}\right) +\omega _1\omega _2 \underline{\widetilde{\gamma }}q\phi _{\not {k}}\right) \end{aligned} \end{aligned}$$

with $$\not { k}={\left\{ \begin{array}{ll}2 {\text{ for }} k=1\\ 1 {\text{ for }} k=2\end{array}\right. }$$.

## Uniqueness

In case the speed of sound *c* is known, reconstruction of $\gamma =\gamma (x)$ in the time domain (1), (2) or the frequency domain formulation (3), (4) amounts to an inverse source problem. Indeed, setting

$$\begin{aligned} \begin{aligned} h(x,\omega _d;\omega _2)&=2\omega _d \nabla {\hat{\phi }}(x,\omega _2+\omega _d)\cdot \overline{\nabla {\hat{\phi }}(x,\omega _2)}\\ m(x,\omega _d;\omega _2)&=2 (\omega _2+\omega _d)\omega _2 \omega _d {\hat{\phi }}(x,\omega _2+\omega _d)\overline{{\hat{\phi }}(x,\omega _2)}\\ \gamma '(x)&=\frac{\gamma (x)-1}{2c(x)^2} \end{aligned} \end{aligned}$$

and multiplying with $c^2$, we can write (4) as

29 $$\begin{aligned} -\omega _d^2 {\hat{\psi }}+{\mathcal {A}}_c {\hat{\psi }}= h(\omega _d;\omega _2)+ m(\omega _d;\omega _2) \gamma ' \end{aligned}$$

Here we denote the difference frequency $\omega _1-\omega _2$ by $\omega _d$ and the solution of (3) with boundary excitation ${\hat{g}}_k={\hat{g}}(\omega )$ by ${\hat{\phi }}_k(\omega )$. Note that the functions *m* and *h* are known from the known excitations ${\hat{g}}$. Moroever, we denote by ${\mathcal {A}}_c$ the elliptic differential operator $-c^2\Delta$ with homogeneous impedance boundary conditions; ${\mathcal {A}}_c$ is a selfadjoint nonnegative definite operator with respect to the weighted $L^2$ inner product with weight function $w=\frac{1}{c^2}$. By $\{(\lambda _k,(\varphi _j^k)_{j\in I_k})\, : \, k\in {\mathbb {N}}\}$ we denote the corresponding eigensystem, where in case of multiple eigenvalues we collect the eigenfunctions corresponding to $\lambda _k$ in the set $\{\varphi _j^k\, : \, j\in I_k\}$ with some finite index set $I_k$. (Note that in one space dimension, the eigenfunctions are single and so $I_k=\{1\}$.) The requirements on *c* for this purpose are

30 $$\begin{aligned} c,\tfrac{1}{c}\in L^\infty (\Omega )\,. \end{aligned}$$

Since the eigenfunctions form an orthonormal basis of $L^2_{w}$, we can expand ${\hat{\psi }}$ with respect to this basis

$$\begin{aligned} {\hat{\psi }}(x,\omega _d;\omega _2) =\sum _{k=1}^\infty \sum _{j\in I_k}\langle {\hat{\psi }}(\omega _d;\omega _2),\varphi _j^k \rangle _{L^2_{w}} \varphi _j^k(x)\,. \end{aligned}$$

This allows us to express the observations according to (5) by

31 $$\begin{aligned} \begin{aligned}&y(x_0,\omega _d;\omega _2)={\hat{\psi }}(x_0,\omega _d;\omega _2) \\&\quad =\sum _{k=1}^\infty \sum _{j\in I_k}\langle {\hat{\psi }}(\omega _d;\omega _2),\varphi _j^k \rangle _{L^2_{w}} \varphi _j^k(x_0) \\&\quad x_0\in \Gamma , \quad \omega _d\in U\,, \end{aligned} \end{aligned}$$

where we assume that we can take observations for all difference frequencies in some set $U$, while $\omega _2$ is fixed. On the other hand, taking inner products of (29) with $\varphi _j^k$ and using the eigenvalue equation ${\mathcal {A}}_c\varphi _j^k=\lambda _j\varphi _j^k$ we obtain the identity

$$\begin{aligned} \begin{aligned}&\langle {\hat{\psi }}(\omega _d;\omega _2),\varphi _j^k\rangle _{L^2_{w}}\\&\quad = \frac{1}{-\omega _d^2+\lambda _j} \langle h(\omega _d;\omega _2)+ m(\omega _d;\omega _2) \gamma ',\varphi _j^k\rangle _{L^2_{w}}. \end{aligned} \end{aligned}$$

Combining this with (31) we get

32 $$\begin{aligned} \begin{aligned}&{\tilde{y}}(x_0,\omega _d;\omega _2)\\&\quad =\sum _{k=1}^\infty \frac{1}{-\omega _d^2+\lambda _k}\sum _{j\in I_k} \langle m(\omega _d;\omega _2) \gamma ',\varphi _j^k\rangle _{L^2_{w}}\varphi _k^j(x_0)\\&\quad x_0\in \Gamma , \quad \omega _d\in U\,, \end{aligned} \end{aligned}$$

where ${\tilde{y}}$ is the modified observation function

$$\begin{aligned} \begin{aligned}&{\tilde{y}}(x_0,\omega _d;\omega _2)\\&\quad =y(x_0,\omega _d;\omega _2)- (-\omega _d^2+{\mathcal {A}}_c)^{-1} h(\omega _d;\omega _2)\,, \end{aligned} \end{aligned}$$

thus a known quantity. In order to obtain from this the desired information on $\gamma '$, we assume that ${\hat{g}}(\omega )$ has been chosen such that *m* factorizes into a frequency dependent and a space dependent part

33 $$\begin{aligned} m(x,\omega _d;\omega _2)=a(\omega _d;\omega _2) b(x) \end{aligned}$$

so that (32) becomes

34 $$\begin{aligned} \begin{aligned}&{\tilde{y}}(x_0,\omega _d;\omega _2)\\&\quad =\sum _{k=1}^\infty \frac{a(\omega _d;\omega _2)}{-\omega _d^2+\lambda _k}\sum _{j\in I_k} \langle b \gamma ',\varphi _j^k\rangle _{L^2_{w}}\varphi _k^j(x_0) \\&\quad x_0\in \Gamma , \quad \omega _d\in U\,. \end{aligned} \end{aligned}$$

Both sides of this equality have sigularities at $\omega _d=\pm \sqrt{\lambda _\ell }$. Thus, these poles provide the location of the eigenvalues of ${\mathcal {A}}_c$ and therewith some information on *c* (see Remark 3 below). Moreover, multiplying with $(\omega _d-\sqrt{\lambda _\ell })$ and taking the limit $\omega _d\rightarrow \sqrt{\lambda _\ell }$, we can extract the contribution due to the $\ell$-th eigenfunction

35 $$\begin{aligned} \begin{aligned}&\lim _{\omega _d\rightarrow \sqrt{\lambda _\ell }} (\omega _d-\sqrt{\lambda _\ell }) {\tilde{y}}(x_0,\omega _d;\omega _2)\\&\quad = -\frac{a(\sqrt{\lambda _\ell };\omega _2)}{2\sqrt{\lambda _\ell }}\sum _{j\in I_k} \langle b \gamma ',\varphi _j^\ell \rangle _{L^2_{w}} \quad x_0\in \Gamma 
\end{aligned} \end{aligned}$$

For this to work out, we need to assume that

36 $$\begin{aligned} \sqrt{\lambda _\ell } \text{ is } \text{ an } \text{ interior } \text{ point } \text{ of } U {\text{ for all }}  \ell \in {\mathbb {N}}. \end{aligned}$$

Finally (35) allows to uniquely determine the coefficients $\langle b \gamma ',\varphi _j^\ell \rangle _{L^2_{w}}$ in

37 $$\begin{aligned} \gamma '(x)=\frac{1}{b(x)} \sum _{\ell =1}^\infty \sum _{j\in I_\ell } \langle b \gamma ',\varphi _j^\ell \rangle _{L^2_{w}} \end{aligned}$$

provided *b* vanishes nowhere and

38 $$\begin{aligned} \{\varphi _j^\ell \vert _\Gamma \,:\, j\in I_\ell \} \text{ is } \text{ linearly } \text{ independent } \end{aligned}$$

Thus we have proven the following uniqueness result on recovery of $\gamma (x)$.

### Theorem 3

Assume that *c* is known and satisfies (30), that $U$ and $\Gamma$ are chosen such that (36), (38) hold, and that ${\hat{g}}(\omega _2+\omega _d)$, is chosen such that (33) holds for all $\omega _d\in U$ with $b\in L^\infty$.

Then $\gamma \in L^2(\Omega )$ is uniquely determined on the set $\{x\in \widetilde{\Omega }\, : \, b(x)\not =0\}$ by the observations $y(x_0,\omega _d;\omega _2)={\hat{\psi }}(x_0,\omega _d;\omega _2)$, $x_0\in \Gamma$, $\omega _d\in U$.

### Remark 1

Obviously, if (36), (38) only hold with ${\mathbb {N}}$ replaced by $\{1,\ldots ,N\}$, we can recover the first *N* coefficients of $b\gamma '$.

Note that no regularity assumptions with respect to $\omega _d$ need to be imposed here.

Condition (38) has been discussed in detail in [14, Remark 4.1]. It is trivially satisfied with $\Gamma$ containing a single point $\{x_0\}$ in one space dimension, since the eigenvalues of ${\mathcal {A}}_c$ are single then. Moroever, it can be extended to higher space dimensions and geometric settings in which the eigenfunctions allow for separation of variables. A simple 2-d example is a disc with radius *r*, where using polar coordinates, the eigenfunctions can be written in terms of Bessel functions. A circle with almost any radius $r_*\in (0,r]$ can then be used as observation manifold $\Gamma$, as shown in [14, Remark 4.1].

To achieve the separability (33) of *m* we supplement the boundary excitation ${\hat{g}}_k(\omega )$ by an interior one $f_{g_k}(\omega )$, which we view as an approximation of a source ${\tilde{g}}(\omega )\,\delta _{\Sigma _k}$ concentrated on $\Sigma _k$, cf., e.g., [15]. The resulting equation for ${\hat{\phi }}_k(\omega )$

$$\begin{aligned} \begin{aligned}&-\omega ^2{\hat{\phi }}_k -\frac{c^2}{d}\Delta {\hat{\phi }}_k = f_{g_k}(\omega ) {\text{ in }} \Omega \\&\partial _\nu {\hat{\phi }}_k=-\sigma _k{\hat{\phi }}_k {\text{ on }} \partial \Omega \setminus \Sigma _k\,,\ \partial _\nu {\hat{\phi }}_k={\hat{g}}_k(\Omega ) {\text{ on }} \Sigma _k \end{aligned} \end{aligned}$$

then has a solution of the form ${\hat{\phi }}_k(x,\omega )={\tilde{a}}(\omega ){\tilde{b}}(x)$ if, e.g., we choose ${\tilde{b}}$ such that $\Delta {\tilde{b}}=0$ in $\Omega$, $\partial _\nu {\tilde{b}}=-\sigma _k{\tilde{b}}$ on $\partial \Omega \setminus \Sigma$, and set ${\hat{g}}_k(\omega ):={\tilde{a}}(\omega )\partial _\nu {\tilde{b}}\vert _\Sigma$, $f_{g_k}(\omega ):=-\omega ^2{\tilde{a}}(\omega ){\tilde{b}}$.

### Remark 2

In case of constant sound speed *c*, uniqueness for the above inverse source problem for $\gamma (x)$ in the time domain formulation (1), (2) from boundary observations under a space-time separability assumption (similar to the space-frequency one (33)) can be concluded from [16, Theorem 7.4.2], provided $\Gamma$, *c* and *T* satisfy [16, condition (1.2.11)], which is basically a condition on sufficient size of $\Gamma$ and *T*, depending on the speed *c* of sound propagation.

Other related uniqueness results for $\gamma$ have been found recently in the context of nonlinearity imaging in [17, 18].

### Remark 3

Note that from the poles on both sides of (34) we also obtain the eigenvalues of ${\mathcal {A}}_c$. According to Sturm-Liouville theory, applied as in [19, Section 5.3], this uniquely determines *c*(*x*) in one space dimension, provided we can take measurements at two different impedance values $\sigma$, ${\tilde{\sigma }}$. Note however, that we need the eigenfunctions of ${\mathcal {A}}_c$ for reconstructing $\gamma '(x)$ according to (37), so this only gives a uniqueness result for *c* alone and no simultaneous uniqueness of *c* and $\gamma$. Also, its restriction to the 1-d setting limits applicability to our experimental setting.

For uniqueness of $c=c(x)$ in higher space dimensions from boundary measurements, results on uniqueness of the space-dependent index of refraction $n(x)=\frac{c_0^2}{c(x)^2}$ in inverse scattering, e.g., [20, Chapter 6] or of the potential in the Schrödinger equation [21, Chapter 5] are relevant. Note however, that *c* appears not only in the equation for the observed quantity $\psi$ but also governs the two excitation wave fields $\phi _1$, $\phi _2$ that enter the $\psi$ equation through a source term. This makes the uniqueness question for *c* more involved than in the mentioned references.

### Remark 4

A proof of unique recovery of both *c* and $\gamma$ is widely open and subject of future research. We will nevertheless in the remainder of this paper discuss some simultaneous numerical reconstruction techniques.

## Iterative reconstruction methods

We return to the general case in which both *c* and $\gamma$ are unknown.

Iteratively regularized Gauss-Newton method IRGNM A regularized Gauss-Newton step for solving (11) defines $$ \left[{\begin{array}{c}\kappa \gamma \end{array}} \right]^{(n+1)}$$ as a minimizer of

$$\begin{aligned} \begin{aligned}&\Vert F( \left[{\begin{array}{c}\kappa \gamma \end{array}} \right]^{(n)})+T^{(n)}( \left[{\begin{array}{c}\kappa \gamma \end{array}} \right]- \left[{\begin{array}{c}\kappa \gamma \end{array}} \right]^{(n)})-y\Vert _{L^2}^2 \\&\quad +\alpha ^{(n)}\Vert ( \left[{\begin{array}{c}\kappa \gamma \end{array}} \right]- \left[{\begin{array}{c}\kappa \gamma \end{array}} \right]^{(0)})\Vert _{L^2}^2 \end{aligned} \end{aligned}$$

where $$T^{(n)}=F'( \left[{\begin{array}{c}\kappa \gamma \end{array}} \right]^{(n)})$$ thus, with $^*$ denoting the Hilbert space adjoint in $L^2$, the Newton step reads as

$$\begin{aligned} \begin{aligned}& \left[{\begin{array}{c}\kappa \gamma \end{array}} \right]^{(n+1)} =  \left[{\begin{array}{c}\kappa \gamma \end{array}} \right]^{(n)}+({T^{(n)}}^* T^{(n)}+\alpha ^{(n)} I)^{-1} \\&\quad \left({T^{(n)}}^* (y-F( \left[{\begin{array}{c}\kappa \gamma \end{array}} \right]^{(n)})+\alpha ^{(n)}( \left[{\begin{array}{c}\kappa \gamma \end{array}} \right]^{(0)}- \left[{\begin{array}{c}\kappa \gamma \end{array}} \right]^{(n)})\right) \end{aligned} \end{aligned}$$

or as $$ \left[{\begin{array}{c}\kappa \gamma \end{array}} \right]^{(n+1)}= \left[{\begin{array}{c}\kappa \gamma \end{array}} \right]^{(n)}+\delta  \left[{\begin{array}{c}\kappa \gamma \end{array}} \right]$$ where $$\delta  \left[{\begin{array}{c}\kappa \gamma \end{array}} \right]$$ solves the variational equation

39 $$\begin{aligned} \begin{aligned}&\langle T^{(n)} \delta  \left[{\begin{array}{c}\kappa \gamma \end{array}} \right], T^{(n)}  \left[{\begin{array}{c}\xi \zeta \end{array}} \right]\rangle + \alpha ^{(n)} \langle \delta  \left[{\begin{array}{c}\kappa \gamma \end{array}} \right], \left[{\begin{array}{c}\xi \zeta \end{array}} \right]\rangle \\&\quad = \langle y-F( \left[{\begin{array}{c}\kappa \gamma \end{array}} \right]^{(n)}), T^{(n)}  \left[{\begin{array}{c}\xi \zeta \end{array}} \right]\rangle + \alpha ^{(n)} \langle  \left[{\begin{array}{c}\kappa \gamma \end{array}} \right]^{(0)}   - \left[{\begin{array}{c}\kappa \gamma \end{array}} \right]^{(n)}  , \left[{\begin{array}{c}\xi \zeta \end{array}} \right]\rangle \\&\quad {\text{ for all }}   \left[{\begin{array}{c}\xi \zeta \end{array}} \right]\in L^2(\widetilde{\Omega })\times L^2(\widetilde{\Omega })\,, \end{aligned} \end{aligned}$$

where $\langle \cdot ,\cdot \rangle$ denotes the $L^2$ inner products

$$\begin{aligned} \begin{aligned}&\langle  \left[{\begin{array}{c}\kappa \gamma \end{array}} \right], \left[{\begin{array}{c}\xi \zeta \end{array}} \right]\rangle =\langle \kappa ,\xi \rangle _{L^2(\widetilde{\Omega })}+\langle \xi ,\zeta \rangle _{L^2(\widetilde{\Omega })},\\&\quad \langle y,z\rangle =\langle y,z\rangle _{L^2(\Gamma )}\,. \end{aligned} \end{aligned}$$

Levenberg-Marquardt method A slightly different version of Newton’s method is the Levenberg-Marquardt method defining the new iterate as a minimizer of

$$\begin{aligned} \begin{aligned}&\Vert F( \left[{\begin{array}{c}\kappa \gamma \end{array}} \right]^{(n)})+T^{(n)}( \left[{\begin{array}{c}\kappa \gamma \end{array}} \right]- \left[{\begin{array}{c}\kappa \gamma \end{array}} \right]^{(n)})-y\Vert _{L^2}^2 \\&\quad +\alpha ^{(n)}\Vert ( \left[{\begin{array}{c}\kappa \gamma \end{array}} \right]- \left[{\begin{array}{c}\kappa \gamma \end{array}} \right]^{(n)})\Vert _{L^2}^2 \end{aligned} \end{aligned}$$

and thus reads as

$$\begin{aligned} \begin{aligned}& \left[{\begin{array}{c}\kappa \gamma \end{array}} \right]^{(n+1)} =  \left[{\begin{array}{c}\kappa \gamma \end{array}} \right]^{(n)}+({T^{(n)}}^* T^{(n)}+\alpha ^{(n)} I)^{-1} \\&\quad {T^{(n)}}^* \left(y-F( \left[{\begin{array}{c}\kappa \gamma \end{array}} \right]^{(n)})\right) \end{aligned} \end{aligned}$$

i.e., as $$ \left[{\begin{array}{c}\kappa \gamma \end{array}} \right]^{(n+1)}= \left[{\begin{array}{c}\kappa \gamma \end{array}} \right]^{(n)}+\delta  \left[{\begin{array}{c}\kappa \gamma \end{array}} \right]$$ with

40 $$\begin{aligned} \begin{aligned}&\langle T^{(n)} \delta  \left[{\begin{array}{c}\kappa \gamma \end{array}} \right], T^{(n)}  \left[{\begin{array}{c}\xi \zeta \end{array}} \right]\rangle + \alpha ^{(n)} \langle \delta  \left[{\begin{array}{c}\kappa \gamma \end{array}} \right], \left[{\begin{array}{c}\xi \zeta \end{array}} \right]\rangle \\&\quad = \langle y-F( \left[{\begin{array}{c}\kappa \gamma \end{array}} \right]^{(n)}), T^{(n)}  \left[{\begin{array}{c}\xi \zeta \end{array}} \right]\rangle \\&\quad {\text{ for all }}   \left[{\begin{array}{c}\xi \zeta \end{array}} \right]\in L^2(\widetilde{\Omega })\times L^2(\widetilde{\Omega })\,. \end{aligned} \end{aligned}$$

Gradient type methods A Landweber step for solving (11) is defined by a gradient descent step for the least squares functional

$$\begin{aligned} \Vert F( \left[{\begin{array}{c}\kappa \gamma \end{array}} \right]^{(n)})-y\Vert _{L^2}^2 \end{aligned}$$

i.e., by

$$\begin{aligned} \begin{aligned}  \left[{\begin{array}{c}\kappa \gamma \end{array}} \right]^{(n+1)}&=  \left[{\begin{array}{c}\kappa \gamma \end{array}} \right]^{(n)}+\mu {T^{(n)}}^* \left(y-F( \left[{\begin{array}{c}\kappa \gamma \end{array}} \right]^{(n)})\right) \end{aligned} \end{aligned}$$

with an appropriately chosen step size $\mu$.

Some remarks on the implementation are in order. For details we refer to, e.g., [13].

**Choice of** $\alpha ^{(n)}$ The regularization parameter in the Newton type methods may be simply chosen along a geometric sequence $\alpha ^{(n)}=c \rho ^n$ for some $c>0$, $\rho \in (0,1)$ in case of the IRGNM versions (both reduced and all-at-once). For the Levenberg-Marquardt method, the choice is somewhat more complicated, namely it has to balance nonlinear and linearized residual in the sense of an inexact Newton method such that

$$\begin{aligned} {\underline{\theta }}\Vert F( \left[{\begin{array}{c}\kappa \gamma \end{array}} \right]^{(n)})-y\Vert \le \text{ res }(\alpha ^{(n)}) \le {\overline{\theta }}\Vert F( \left[{\begin{array}{c}\kappa \gamma \end{array}} \right]^{(n)})-y\Vert \end{aligned}$$

for some constants $0<{\underline{\theta }}<{\overline{\theta }}<1$, where $$\text{ res }(\alpha )=\Vert F( \left[{\begin{array}{c}\kappa \gamma \end{array}} \right]^{(n)})+T^{(n)}( \left[{\begin{array}{c}\kappa \gamma \end{array}} \right]^{(n+1)}(\alpha )- \left[{\begin{array}{c}\kappa \gamma \end{array}} \right]^{(n)})-y\Vert $$, cf. [22].

Stopping rule To avoid unbounded propagation of the measurement noise through the iterations, the methods defined above have to be stopped at an appropriate index *n*. A widely used and well-investigated method for this is the discrepancy principle, which for a given noise level $\delta$ and a safety factor $\tau >1$ defines $n=n_*=n_*(\delta )$ as the first index such that

$$\begin{aligned} \Vert F( \left[{\begin{array}{c}\kappa \gamma \end{array}} \right]^{(n)})-y\Vert \le \tau \delta \,. \end{aligned}$$

Multiple observations As we have seen in Section 4, unique recovery of even just one of the two coefficients *c* and $\gamma$ requires boundary measurements for several frequencies - a fact that is evident from a simple dimension count. Also the fact that the focal point where the high frequency beams interact is moved through the region of interest should be taken into account by incorporating multiple excitations. This corresponds to using Neumann conditions $g_k^\ell$ at transducer locations $\Sigma _k^\ell$ for $\ell \in \{1,\ldots ,L\}$. Finally, several receiver array locations $\Gamma ^m$, $m\in \{1,\ldots ,M\}$, might be used to recover a single pair of $\kappa$ and $\gamma$. Thus, we actually deal with a set of several model and observation operators $A^\ell$, $\ell \in \{1,\ldots ,L\}$, $C^m$, $m\in \{1,\ldots ,M\}$ respectively. Labelling the resulting forward operators $F_p=C^m \circ S^\ell$ and data $y_p$ for $p=(m-1)L+\ell$, we can write the inverse problem of reconstructing $$ \left[{\begin{array}{c}\kappa \gamma \end{array}} \right]$$ as a system of operator equations

41 $$\begin{aligned} F_p( \left[{\begin{array}{c}\kappa \gamma \end{array}} \right])=y_p \, \quad p\in \{1,\ldots ,P=L\cdot M\} \end{aligned}$$

and apply Kaczmarz type methods as follows:

- parallelly apply one step of an iterative reconstruction method to each of the equations in (41) and then combine the resulting reconstructions $$ \left[{\begin{array}{c}\kappa \gamma \end{array}} \right]_p$$ in a proper way, e.g.,
  42 $$\begin{aligned} \begin{aligned}  \left[{\begin{array}{c}\kappa \gamma \end{array}} \right]_p^{(n+1)}&= \left[{\begin{array}{c}\kappa \gamma \end{array}} \right]^{(n)}   +G_p( \left[{\begin{array}{c}\kappa \gamma \end{array}} \right]^{(n)}   ,F_p,y_p),\ p=1,\ldots P, \\  \left[{\begin{array}{c}\kappa \gamma \end{array}} \right]^{(n+1)}&=\frac{1}{P}\sum _{p=1}^P  \left[{\begin{array}{c}\kappa \gamma \end{array}} \right]_p^{(n+1)} \end{aligned}\end{aligned}$$
- sequentially perform one step of an iterative reconstruction method in a cyclically repeated manner
  43 $$\begin{aligned}  \left[{\begin{array}{c}\kappa \gamma \end{array}} \right]^{(n+1)}= \left[{\begin{array}{c}\kappa \gamma \end{array}} \right]^{(n)}+G_p( \left[{\begin{array}{c}\kappa \gamma \end{array}} \right]^{(n)},F_p,y_p) \end{aligned}$$
  where $p=\text {mod}(n-1,P)+1$, (the order in which the indices *p* are addressed could as well be randomized).

In here, $$G_p( \left[{\begin{array}{c}\kappa \gamma \end{array}} \right]^{(n)},F_p,y_p)$$ is defined by one of the Newton or gradient steps defined above.

Algorithms For a pseudocode description of the methods discussed above, see the tables in the Appendix of [23].

## Outlook

In this paper we have made some first steps towards putting the problem of vibroacoustic imaging into the mathematical framework of inverse problems and regularization. We have presented a model in frequency domain, proven uniqueness of recovery of the spatially varying nonlinearity parameter $\gamma (x)$ from pressure measurements at multiple frequencies, and derived Newton as well as gradient based reconstruction methods.

The numerical effort of the of the devised numerical methods has to be assessed with respect to number of steps in terms of the noise level $\delta$ ($n_*(\delta )=O(\log (1/\delta ))$ for Newton type and $n_*(\delta )=O(1/\delta ^2)$ for gradient type) and effort per step (one solve of (3), (4) and one of its linearization in each gradient step; and latter also for each Jacobi matrix – vector multiplication when iteratively computing a Newton step). A more concrete comparison by means of a numerical implementation is subject of future work.

An analysis of noise propagation and convergence as the noise level $\delta$ tends to zero with the above mentioned choices of regularization parameters and stopping indices follows, e.g., from the results in [13] and the references therein, provided the forward operator and the smoothness of the initial error satisfy the conditions stated there, which are yet to be established.

Among important analytical questions yet to be answered, there is uniqueness of simultaneous reconstruction of *c*(*x*) and $\gamma (x)$. To this end, the use of multiple excitation locations (instead of or in addition to multiple frequencies), corresponding to shifting the focus of the interacting high-frequency beams around the region of interest, needs to be further investigated. Moreover, a priori information should be taken into account. Indeed, an important special case is the one of piecewise constant coefficients, in which only the shapes of finitely many subdomains and finitely many values of *c* and $\gamma$ are to be found: Here one would expect uniqueness even from boundary data at just a few frequencies, resulting from appropriately chosen excitations.

A computational framework for the reconstruction of piecewise constant coefficients could be based on the by now standard approach of alternatingly recovering the support and the value of inclusions in a homogeneous background. For a simultaneous recovery of both support and value, the known advantages of total variation regularization can be made use of. In case of known parameter values, also regularization by bound constraints (using the known values as bounds) is a promising approach [24].

Concerning forward simulation, we point to the fact that the high frequency waves $\phi _1$, $\phi _2$ have a strongly preferred direction of propagation, which can justify the use of a parabolic approximation, cf., e.g., [25]. Indeed, for efficient numerical simulation a decomposition approach has been devised in [7, 8] that splits the forward problem into a three components: (a) directed high frequency propagation of the two beams described by $\phi _1$, $\phi _2$, (b) nonlinear interaction of these at the focal point, and (c) undirected low frequency propagation to the measurement array via $\psi$. This could also be implemented in our framework; the adjoint equations for Landweber iteration would have to be re-derived for this purpose.

Also the model itself might have to be modified. Besides the use of a parabolic approximation in phase (a), also fractional damping e.g., [19, 26] is relevant in ultrasonics.
